# Adhesive Bonding to Hydraulic Gel-Forming Calcium Silicate-Based Cements: Effects of Cement Type, Adhesive Strategy, and Restoration Timing

**DOI:** 10.3390/gels12080748

**Published:** 2026-08-20

**Authors:** Gizem Akın Tartuk, Merve Yeniçeri Özata, Sadullah Kaya

**Affiliations:** Department of Endodontics, Faculty of Dentistry, Dicle University, Diyarbakır 21280, Turkey

**Keywords:** adhesive strategy, calcium silicate hydrate (C-S-H) gel, gel maturation, hydraulic calcium silicate-based cement, shear bond strength, Well-Root PT

## Abstract

Calcium silicate-based cements are hydraulic biomaterials that form a calcium silicate hydrate (C-S-H) gel during hydration, a process fundamental to their setting and progressive physicochemical development. Although these materials are widely used in vital pulp therapy, evidence regarding the effects of adhesive strategy and restoration timing on bonding performance, particularly for newer premixed formulations, remains limited. This study evaluated the effects of cement type, adhesive strategy, and restoration timing on the shear bond strength (SBS) of resin composite bonded to MTA Angelus and Well-Root PT. A total of 270 specimens were distributed across two cement types, three adhesive strategies, and three restoration intervals (45 min, 24 h, and 7 days). After thermocycling, SBS and failure modes were assessed. SBS data were analyzed using three-way ANOVA, whereas failure-mode distributions were evaluated using chi-square or Fisher’s exact tests (α = 0.05). Cement type, adhesive strategy, restoration timing, and their interactions significantly influenced SBS (*p* < 0.001). Well-Root PT exhibited higher early bond strength than MTA Angelus. The two-step etch-and-rinse adhesive produced the highest SBS values, whereas the universal adhesive produced the lowest. Restoration after 24 h significantly increased SBS compared with restoration after 45 min, with no additional improvement observed at 7 days. Within the limitations of this in vitro study, bonding performance was influenced by cement type, adhesive strategy, and restoration timing. The time-dependent increase in SBS is consistent with continued hydration and development of the C-S-H gel-based cement matrix, although the specific microstructural changes underlying this behavior were not directly characterized.

## 1. Introduction

Preservation of pulp vitality is a central objective of biologically oriented and minimally invasive dental treatment. Vital pulp therapy (VPT), including indirect and direct pulp capping and pulpotomy, is intended to maintain the function of compromised or exposed pulp tissue after carious, traumatic, or iatrogenic injury. Successful treatment, however, depends on more than the healing capacity of the pulp–dentin complex [1]. An effective coronal seal is equally important because limiting bacterial re-entry helps maintain the conditions required for pulpal repair and long-term treatment stability [2].

Hydraulic calcium silicate-based cements (CSCs) have become integral to VPT because their biological activity is coupled with a moisture-dependent setting reaction. During hydration, calcium silicate hydrate (C-S-H) gel develops as the principal binding phase and progressively contributes to the structure and physicochemical behavior of the hardened cement. The state of this evolving matrix is also relevant to restorative procedures because adhesive materials are applied directly to the hydrated cement surface. CSCs additionally exhibit favorable biocompatibility, bioactivity, sealing characteristics, and calcium ion release associated with mineralization processes [1,3]. Mineral Trioxide Aggregate (MTA), introduced in the early 1990s [3], remains the reference material for this class and has been used in perforation repair, root-end filling procedures, apexogenesis, apexification, pulp capping, and pulpotomy [4]. Its clinical versatility is supported by its ability to set under moist conditions, hard-tissue inductive potential, sealing capacity, alkaline environment, and calcium hydroxide release [4,5].

The two cements evaluated in the present study represent different approaches to hydraulic cement formulation and handling. MTA Angelus (Angelus, Londrina, Brazil) is a powder–liquid MTA formulation in which the absence of calcium sulfate contributes to a considerably shorter setting time than that of conventional MTA formulations [6,7]. Its major constituents include tricalcium silicate, dicalcium silicate, tricalcium aluminate, and bismuth oxide. Although the shorter setting period may facilitate earlier restorative procedures, manual powder–liquid proportioning and mixing remain potential sources of handling variability, and the resulting cement surface differs substantially from dentine as an adhesive substrate [8,9]. Well-Root PT (Vericom Co., Chuncheon, Republic of Korea), in contrast, is supplied as a ready-to-use calcium aluminosilicate-based cement that hardens after exposure to moisture. Available manufacturer information and laboratory data indicate an initial setting time of approximately 5 min and a final setting time of approximately 45 min. Its premixed presentation avoids operator-dependent powder–liquid proportioning and simplifies material delivery. Laboratory investigations have also reported favorable biocompatibility, bioactivity, calcium ion release, and mineralization-related properties for Well-Root PT [10,11]. Nevertheless, information regarding its bonding to resin-based restorative materials remains limited, particularly in direct comparison with established MTA formulations across different adhesive strategies and restoration intervals [8,12,13].

Establishing a stable restorative interface on these materials is challenging because a hydrated CSC surface is fundamentally different from dentine. Resin composites are commonly placed over CSCs because of their esthetic and mechanical properties and their compatibility with adhesive restorative procedures [12,14], yet the substrate encountered by the adhesive is mineral-rich, alkaline, moisture-dependent, and continuously affected by hydration. C-S-H gel, calcium hydroxide, residual unhydrated particles, and surface porosity may all contribute to the characteristics of this interface [14,15,16]. Consequently, adhesive wetting and interaction with a CSC cannot be assumed to follow the same behavior observed on the collagen-containing dentine substrate.

The interaction between an adhesive system and a calcium silicate-based cement is likely to depend on both the conditioning approach and the characteristics of the cement surface. Etch-and-rinse protocols introduce a separate phosphoric acid treatment, whereas self-etch approaches rely on acidic functional monomers for simultaneous conditioning and substrate interaction; universal adhesives provide additional flexibility in application mode [17]. These distinctions may become particularly important on calcium silicate-based substrates, whose mineral-rich and moisture-dependent surfaces differ substantially from dentine and may respond differently to adhesive conditioning [14,15]. Accordingly, previous studies have reported variable bonding outcomes depending on the cement formulation, adhesive approach, and experimental conditions [12,14,16,18,19]. Etch-and-rinse strategies have shown favorable performance for several MTA-based materials [12], although this advantage has not been consistently demonstrated across all calcium silicate-based cements [14]. Such material-dependent variability supports further evaluation of adhesive protocols for newer premixed formulations, including Well-Root PT.

Restoration timing represents another potentially important determinant of bonding. Hydration of hydraulic CSCs continues beyond initial hardening, with corresponding evolution of the cement matrix and surface characteristics. Thus, the substrate available for adhesive application shortly after placement may differ from that encountered after a longer hydration period. Previous bond-strength studies support a material-dependent effect of restoration timing. Sulwińska et al. reported higher bonding values when composite resin was applied to MTA after 24 h rather than immediately [16], whereas Celiksoz and Irmak found that the manufacturer-recommended interval was adequate for Biodentine but that bonding to RetroMTA improved with longer waiting periods [20]. These observations suggest that the influence of time cannot be generalized across different CSC formulations. Progressive development of the C-S-H gel-based matrix provides a plausible materials-based context for such changes, although the relationship between gel maturation and adhesive performance requires direct microstructural characterization [13,20].

Despite increasing interest in adhesive restoration over hydraulic CSCs, MTA Angelus and Well-Root PT have not been directly compared within a single factorial design incorporating both adhesive strategy and restoration timing. The associated distribution of failure modes at these interfaces also remains insufficiently characterized. Therefore, the present study evaluated the effects of cement type, adhesive strategy, and restoration timing on the shear bond strength (SBS) of resin composite bonded to MTA Angelus and Well-Root PT and assessed the corresponding failure patterns. By examining these factors simultaneously, the study aimed to provide a more integrated assessment of bonding to hydraulic gel-forming calcium silicate-based biomaterials.

The null hypotheses were that cement type, adhesive strategy, and restoration timing would not significantly affect SBS and that no significant interactions would occur among these factors.

## 2. Results and Discussion

### 2.1. Shear Bond Strength

**Figure 1:**
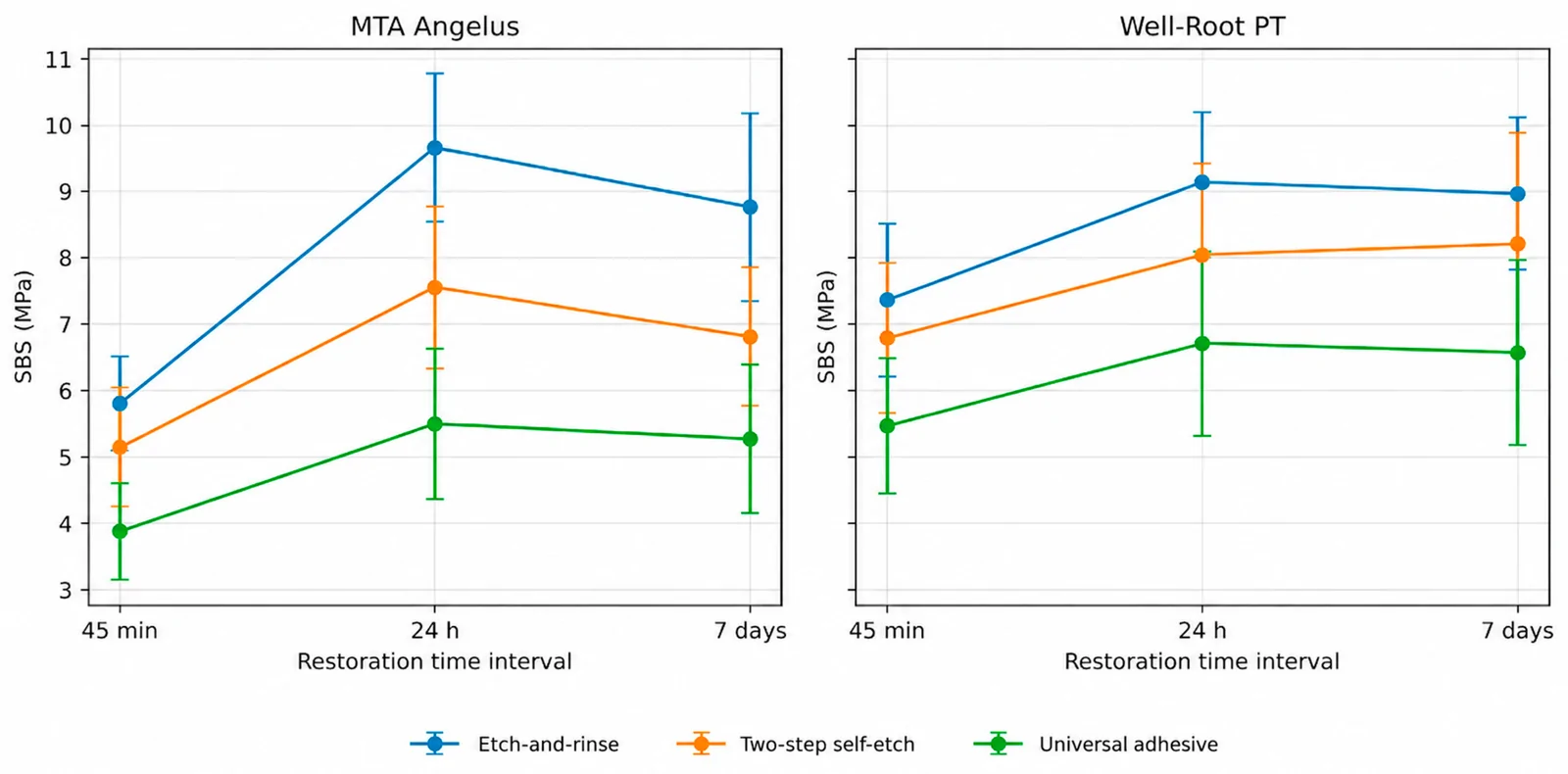
Interaction plot showing the effects of adhesive strategy and restoration time interval on the shear bond strength (SBS) of MTA Angelus and Well-Root PT. Data are presented as mean ± standard deviation (SD). Error bars indicate standard deviations.

**Figure 2:**
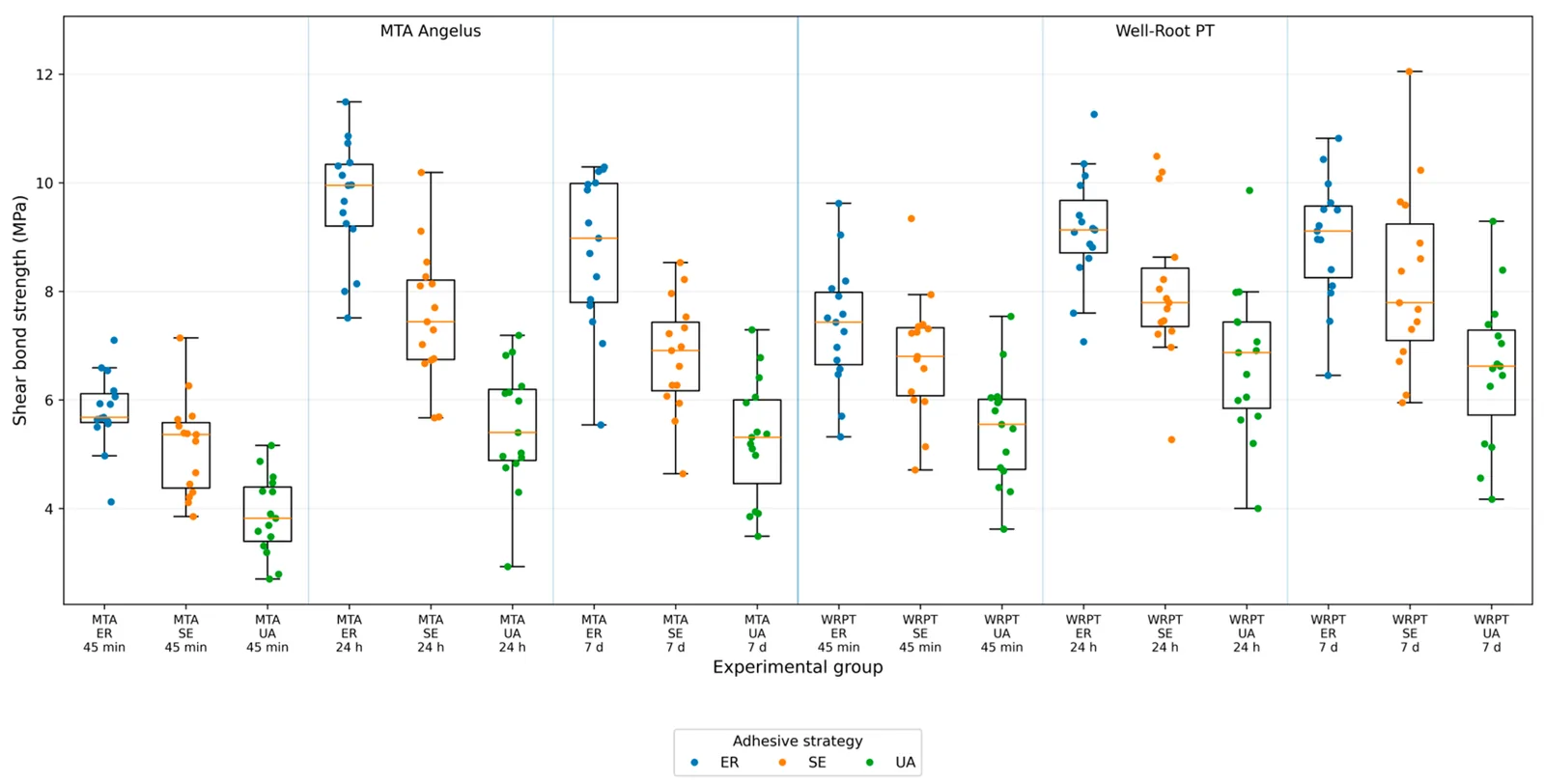
Specimen-level distribution of shear bond strength (SBS) values according to cement type, adhesive strategy, and restoration interval. Box plots represent the median and interquartile range, with whiskers indicating data dispersion; overlaid points represent individual specimen measurements (*n* = 15 per experimental group). ER, two-step etch-and-rinse; SE, two-step self-etch; UA, universal adhesive applied in self-etch mode; MTA, MTA Angelus; WRPT, Well-Root PT.

The assumptions for parametric analysis were satisfied, with normally distributed SBS data (Shapiro–Wilk, *p* > 0.05) and homogeneous variances (Levene’s test, F = 0.956, *p* = 0.509). Three-way ANOVA revealed that cement type, adhesive strategy, restoration timing, and all interaction terms significantly influenced SBS (*p* < 0.001; Table 1). The interaction between adhesive strategy and restoration timing for each calcium silicate-based cement is presented in Figure 1. Mean SBS values for all experimental groups are summarized in Table 2, while the specimen-level distributions of SBS values are illustrated in Figure 2.

#### 2.1.1. Effect of Cement Type

Well-Root PT generally exhibited higher SBS values than MTA Angelus, particularly at the 45 min restoration interval, despite the significant interactions observed among the experimental factors. The differences were most evident when the two-step etch-and-rinse and two-step self-etch adhesives were used, whereas the SBS values of the two calcium silicate-based cements became comparable after 24 h and 7 days, particularly with the etch-and-rinse adhesive.

The material-dependent differences observed during the early restorative period may be related, at least in part, to differences in formulation and handling characteristics between the two cements. Well-Root PT is supplied as a premixed material, thereby eliminating the powder–liquid proportioning and manual mixing required for MTA Angelus [10,11]. The physicochemical properties of MTA Angelus have been shown to be sensitive to variations in the powder-to-water ratio, including changes in setting time, pH, calcium ion release, and solubility [21]. Accordingly, eliminating this operator-dependent mixing step may contribute to more consistent early substrate characteristics.

Early surface moisture may also have contributed to the difference observed at 45 min. Although all specimens were maintained under identical humid conditions and excess surface moisture was removed using the same blot-drying protocol before adhesive application, these standardized procedures do not necessarily imply identical surface–water interactions for the two cements. Material-specific wettability and water absorption behavior have been reported for Well-Root PT in comparison with other hydraulic calcium silicate cements [22], supporting the possibility that differences in surface–water interaction may influence the characteristics of the early bonding substrate. However, surface moisture was not quantitatively assessed in the present study, and the available wettability data do not directly compare MTA Angelus and Well-Root PT at the 45 min interval. Therefore, the higher early SBS of Well-Root PT cannot be attributed exclusively to its premixed formulation; differences in early surface moisture may also have contributed, although the relative contribution of these factors cannot be determined from the present data.

#### 2.1.2. Effect of Adhesive Strategy

The choice of adhesive strategy exerted a significant influence on shear bond strength (SBS) across all experimental groups (*p* < 0.001). In general, the two-step etch-and-rinse adhesive achieved the highest bond strength, followed by the two-step self-etch adhesive, while the universal adhesive consistently showed the lowest SBS. Post hoc analysis revealed that the etch-and-rinse strategy significantly outperformed the universal adhesive across all cements and time intervals (*p* < 0.05). Similarly, the two-step self-etch adhesive yielded significantly higher SBS results than the universal adhesive in almost all instances, with the sole exception of the MTA Angelus group at 45 min (*p* = 0.099). While significant differences between the etch-and-rinse and self-etch adhesives were noted for MTA Angelus at 24 h and 7 days (*p* < 0.05), no such differences were found for Well-Root PT at any interval.

The superior performance of the two-step etch-and-rinse adhesive may primarily be related to the separate phosphoric acid conditioning step. Phosphoric acid conditioning has been reported to alter the surface characteristics of MTA [15] and to improve its wettability and bond strength to resin composite [18], potentially favoring subsequent adhesive interaction. This interpretation is consistent with the systematic review and meta-analysis by Hardan et al., which showed that etch-and-rinse strategies generally provide higher bond strength to MTA than self-etch approaches [14]. Similarly, Naiboğlu et al. reported higher SBS to MTA Angelus when a universal adhesive was applied in etch-and-rinse rather than self-etch mode and demonstrated acid-induced morphological alterations of the cement surface by SEM–EDS analysis [12].

In contrast, the two-step self-etch and universal adhesive strategies used in the present study did not include a separate phosphoric acid conditioning step and relied on acidic functional monomers for substrate interaction. The consistently lower SBS obtained with the universal adhesive is consistent with previous reports showing that bonding performance to MTA may be lower with self-etch or simplified approaches than with etch-and-rinse strategies [12,16]. However, the differences among the adhesive systems should not be attributed solely to the etching strategy, because adhesive-specific factors such as functional monomers, solvent type, filler content, and other formulation characteristics may also influence bonding to calcium silicate-based materials [12,14]. Therefore, the present findings suggest that both surface-conditioning strategy and adhesive-specific formulation characteristics may contribute to bonding performance on these substrates.

Because G-Premio Bond was examined only in self-etch mode, the present results should not be interpreted as indicating inherently lower performance of universal adhesives irrespective of application strategy. Previous studies have reported improved bonding to MTA-based substrates when universal adhesives were preceded by phosphoric acid conditioning [12,23]. Thus, application of G-Premio Bond using an etch-and-rinse approach could potentially yield different outcomes, particularly for MTA Angelus. However, this possibility cannot be confirmed from the present data because the two application modes were not directly compared, and corresponding evidence for Well-Root PT remains limited. Future studies should therefore compare self-etch and etch-and-rinse application of the same universal adhesive.

#### 2.1.3. Effect of Restoration Time Interval

Restoration timing significantly influenced SBS across all experimental groups (*p* < 0.001). The 45 min groups consistently exhibited the lowest bond strength values regardless of cement type or adhesive strategy. In contrast, specimens restored after 24 h and 7 days demonstrated significantly higher SBS values than those restored after 45 min (*p* < 0.05), whereas no significant differences were observed between the 24 h and 7-day groups (*p* > 0.05). For Well-Root PT, SBS increased from 45 min to 24 h and subsequently remained stable, whereas MTA Angelus exhibited a similar trend, with the highest SBS recorded after 24 h.

The marked increase in SBS from 45 min to 24 h indicates that the bonding substrate continued to evolve after the initial setting period. This time-dependent behavior is compatible with the hydration characteristics of hydraulic calcium silicate-based cements, in which the C-S-H gel-based matrix develops progressively beyond initial hardening [24]. Continued hydration is accompanied by changes in the physicochemical and structural characteristics of the cement, and maturation of the substrate has previously been associated with differences in subsequent adhesive interaction [25]. Time-dependent surface changes have also been reported for Well-Root PT during continued exposure to an aqueous environment, further illustrating the dynamic nature of the hydrated cement surface [22].

From this perspective, the lower SBS recorded at 45 min may reflect bonding to a comparatively early-stage hydrated substrate, whereas the higher values obtained after 24 h may be associated with further development and stabilization of the cement matrix and surface. The absence of an additional increase between 24 h and 7 days suggests that, under the conditions of the present study, the changes most relevant to adhesive performance occurred predominantly during the first 24 h. Although progressive development of the C-S-H gel network provides a plausible materials-based framework for this behavior, the present SBS data alone do not establish a direct relationship between gel maturation and bond strength. Accordingly, the time-dependent bonding pattern is interpreted in the context of previously described hydration phenomena rather than as direct evidence of specific microstructural changes.

A similar time-dependent bonding pattern has been reported in previous studies. Sulwińska et al. reported the highest bond strength to MTA after a 24 h restoration interval [16], while Palma et al. demonstrated that restoration timing may influence bonding to hydraulic calcium silicate-based materials [2,13]. In contrast, Celiksoz and Irmak reported that the manufacturer-recommended restoration time was adequate for Biodentine, whereas RetroMTA showed improved bonding with longer waiting periods [20]. Collectively, these findings indicate that the influence of restoration timing is material-dependent and that a single optimal delay cannot be generalized to all calcium silicate-based cements.

#### 2.1.4. Failure Mode Distribution

**Figure 3:**
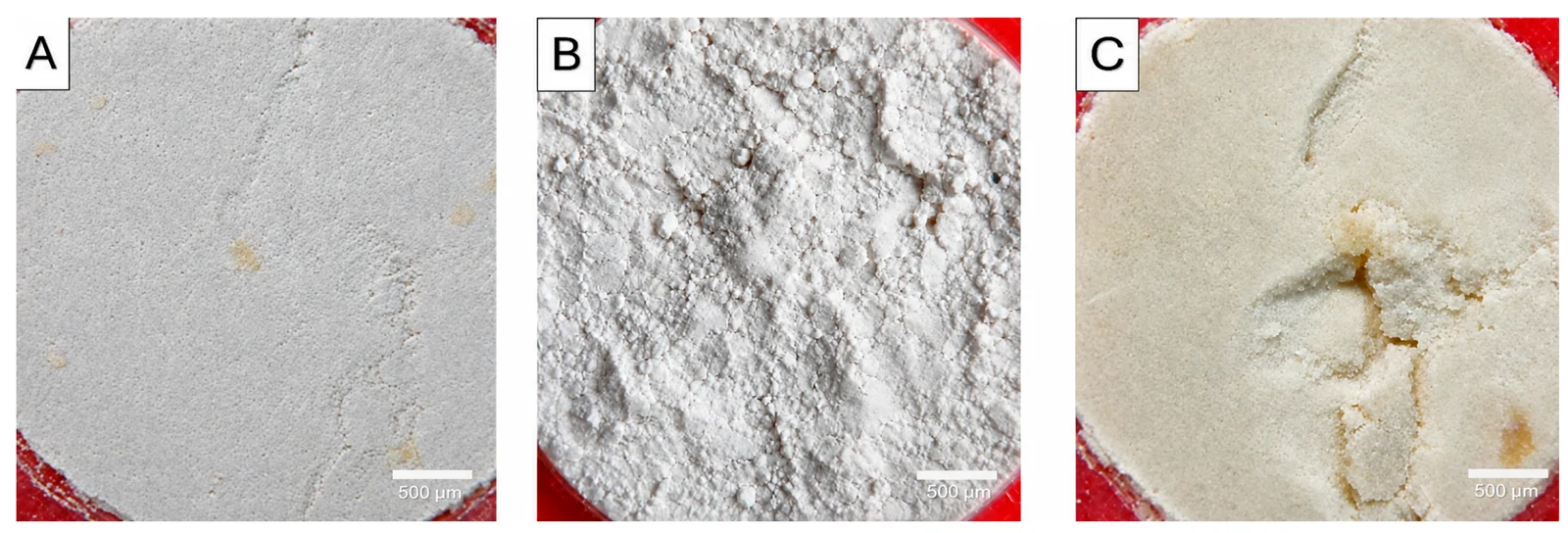
Representative stereomicroscopic images of the failure patterns observed after shear bond strength testing at ×40 magnification: (**A**) adhesive failure at the cement–resin composite interface; (**B**) cohesive failure within the calcium silicate-based cement; and (**C**) mixed failure showing both adhesive and cohesive components.

Chi-square analysis demonstrated a significant association between adhesive strategy and failure mode distribution (χ^2^ = 15.69, df = 4, *p* = 0.004), whereas no significant difference was observed between MTA Angelus and Well-Root PT (χ^2^ = 3.21, df = 2, *p* = 0.201). Adhesive failures predominated in specimens bonded with the universal adhesive, particularly at the 45 min restoration interval. In contrast, cohesive and mixed failures became more frequent in the etch-and-rinse groups and after delayed restoration (24 h and 7 days). Failure mode distributions for all experimental groups are presented in Table 3, and representative stereomicroscopic images of adhesive, cohesive, and mixed failure patterns are shown in Figure 3.

The failure mode distribution showed a trend that paralleled the SBS findings, with adhesive failures occurring more frequently in groups with lower bond strength, particularly those restored after 45 min and bonded with the universal adhesive. Conversely, cohesive and mixed failures were more frequently observed in groups showing higher SBS values. However, failure patterns should be regarded as complementary rather than confirmatory evidence of interfacial bonding performance. In shear bond strength testing, the location and pattern of fracture may be influenced not only by the strength of the bonded interface but also by stress concentration, specimen geometry, loading configuration, and the cohesive strength of the substrates [26,27]. Therefore, the greater frequency of cohesive or mixed failures in the higher-SBS groups cannot, by itself, be interpreted as direct evidence of superior interfacial integrity.

No significant difference in failure mode distribution was detected between MTA Angelus and Well-Root PT despite the higher early SBS values observed for Well-Root PT. Although stereomicroscopic examination at ×40 magnification allowed standardized classification of the fractures as adhesive, cohesive, or mixed, it did not permit detailed characterization of microscopic crack propagation, residual material at the interface, or fine transitions between adhesive and cohesive fracture regions. Consequently, the apparent relationship between SBS and failure mode in the present study should be interpreted cautiously. Higher-resolution fractographic examination using SEM would have provided more definitive information regarding the morphology and integrity of the fractured cement–adhesive interface.

### 2.2. Overall Interpretation and Clinical Implications

The significant three-way interaction among cement type, adhesive strategy, and restoration timing indicates that the bonding performance of resin composite to calcium silicate-based cements cannot be optimized by considering any single factor in isolation. Instead, these variables should be evaluated collectively when selecting an adhesive strategy. Among the combinations investigated, Well-Root PT restored after 24 h using the two-step etch-and-rinse adhesive provided one of the most favorable bonding conditions, whereas MTA Angelus restored after 45 min with the universal adhesive represented the least favorable combination. These findings demonstrate that the relative performance of an adhesive protocol is both material- and time-dependent and cannot be attributed to a single mechanistic factor. Accordingly, all null hypotheses were rejected.

The variability observed in the present study is consistent with previous reports demonstrating that bond strength is influenced by the calcium silicate-based cement, adhesive strategy, restorative material, and restoration timing [14,28,29]. This variability may partly reflect the fact that contemporary adhesive strategies were primarily developed for dentine, which differs substantially from calcium silicate-based cements in terms of composition, hydration behavior, alkalinity, moisture content, and surface chemistry. Consequently, adhesive strategies that perform predictably on dentine cannot be directly extrapolated to calcium silicate-based materials [14,15]. Furthermore, all specimens in the present study were subjected to 5000 thermocycles before bond strength testing. Thermocycling exposes bonded interfaces to repeated temperature-related dimensional changes [30] and has been shown to reduce bonding performance at calcium silicate cement–resin interfaces [31]. Thus, the SBS values obtained in the present study reflect adhesive performance after a standardized thermal aging challenge rather than immediate bonding alone. More extensive thermal aging may lead to further degradation of the bonded interface and lower absolute SBS values. Extended thermocycling studies on dentine have shown that adhesive-dependent differences can persist with increasing numbers of thermal cycles, with a conventional two-step self-etch adhesive maintaining higher bond strength than universal adhesives under the tested degradation conditions [32]. This suggests that some of the strategy-dependent differences observed in the present study may persist during more prolonged aging; however, the exact relative performance of the three adhesive approaches cannot be predicted from the available evidence. Aging behavior depends on both the adhesive system and the bonded substrate, and findings obtained from dentine cannot be directly extrapolated to hydraulic calcium silicate-based cements. Therefore, extended aging protocols incorporating higher numbers of thermocycles, together with mechanical fatigue and long-term aqueous storage, would be valuable for determining the long-term stability of the adhesive interfaces evaluated in the present study.

The use of acrylic blocks should also be considered when interpreting the clinical relevance of the present findings. This model was selected to provide standardized specimen dimensions and reproducible testing geometry, thereby minimizing substrate-related variability and allowing the effects of cement type, adhesive strategy, and restoration timing to be examined under controlled conditions. Nevertheless, this degree of standardization does not reproduce the complex biological environment of natural dentine. Dentinal tubule orientation has been shown to influence adhesive bonding to dentine [33], while dentinal fluid movement under simulated pulpal pressure may further affect interfacial bonding performance [34]. In addition, calcium silicate-based cements can interact directly with dentine through ion exchange and mineralization-related changes at the cement–dentine interface [35], phenomena that cannot be reproduced when the cement is supported by an acrylic substrate. Therefore, the absence of dentinal permeability, tubular architecture, and substrate heterogeneity in the present model may have reduced clinically relevant sources of variability and excluded potential cement–dentine interactions. Accordingly, the SBS values obtained in this study should primarily be interpreted as comparative measures of the cement–adhesive interface under standardized laboratory conditions rather than as direct estimates of clinical bond strength. Future studies using natural dentine, simulated pulpal pressure, and clinically representative cavity configurations are warranted to determine whether the relative differences observed among the tested materials, adhesive strategies, and restoration timings are maintained under more clinically relevant conditions.

From a clinical perspective, the higher SBS values obtained after 24 h should be balanced against the practical advantages of completing vital pulp therapy and definitive restoration in a single appointment. Although delaying restoration for 24 h provided more favorable bonding conditions than restoration after 45 min in the present study, these in vitro data do not establish whether the magnitude of this improvement is sufficient to justify an additional patient visit. In particular, no clinically validated SBS threshold is currently available for the cement–composite interface that would allow the 45 min values to be classified as either clinically adequate or inadequate. The development of faster-setting calcium silicate cements has specifically increased interest in single-visit restorative protocols, and recent laboratory studies have demonstrated that resin-based restorations can be bonded to fast-setting calcium silicate cements shortly after placement, although the immature mechanical properties of the cement remain an important consideration during early restoration [31,36]. Therefore, when a second appointment is clinically feasible and maximizing interfacial bond strength is prioritized, a 24 h delay may be advantageous based on the present findings. Conversely, when completion of treatment in a single visit is desirable, restoration after the initial setting period may remain a reasonable clinical option, particularly with a material showing more favorable early bonding performance such as Well-Root PT. This interpretation should nevertheless remain cautious, because the present study evaluated laboratory SBS rather than clinical restoration survival. Accordingly, the choice between early and delayed restoration should reflect both material-specific bonding behavior and patient- and treatment-related considerations rather than restoration timing alone.

### 2.3. Study Limitations

Despite the strengths of the present study, several limitations should be considered when interpreting the findings. First, the study was conducted under in vitro conditions and therefore could not fully reproduce the biological, mechanical, and environmental complexities of the clinical setting. Second, although thermocycling was performed to simulate thermal fluctuations encountered in the oral environment, other aging factors, including mechanical fatigue loading, enzymatic degradation, and long-term water storage, were not evaluated. Consequently, the long-term durability of the adhesive interfaces under complex clinical conditions remains uncertain. Third, specimens were prepared using standardized acrylic blocks rather than natural dentine. Although this approach reduced substrate-related experimental variability, it excluded dentinal permeability, tubular architecture, pulpal fluid-related moisture, and direct cement–dentine interactions, as discussed above. Consequently, the present findings require validation in dentine-based models before broader clinical extrapolation. The physicochemical evolution of the cement substrates was not characterized at the individual restoration intervals; therefore, the relationship between hydration-related changes in the C-S-H gel-based matrix and the observed time-dependent bonding behavior remains to be clarified through complementary microstructural and phase analyses. In addition, failure mode analysis was based solely on stereomicroscopic examination because SEM and EDX analyses were not performed to characterize the fractured surfaces and interfacial morphology. Finally, only one resin composite material and a limited number of adhesive strategies were evaluated, and the universal adhesive was tested exclusively in self-etch mode. Therefore, the present findings do not allow direct comparison of self-etch and etch-and-rinse application modes within the same universal adhesive. Future studies should incorporate natural dentine substrates, more comprehensive artificial aging protocols, and advanced surface characterization techniques to further elucidate the mechanisms governing adhesion to calcium silicate-based cements and to determine the long-term clinical relevance of these findings.

## 3. Conclusions

Within the limitations of this in vitro study, the bonding performance of resin composite to calcium silicate-based cements was significantly influenced by cement type, adhesive strategy, restoration timing, and the interactions among these factors. The findings indicate that adhesive performance depends on the combined effects of material characteristics, surface conditioning, and the progressive maturation of the calcium silicate hydrate (C-S-H) gel network rather than on any single variable. Premixed Well-Root PT demonstrated more favorable early bonding performance than MTA Angelus, while the two-step etch-and-rinse adhesive provided the most reliable bonding protocol. Delaying restoration for 24 h significantly improved bond strength compared with restoration after 45 min, whereas no additional benefit was observed after 7 days. However, this laboratory advantage should not be interpreted as requiring a second clinical visit, because the present study was not designed to establish a clinically acceptable SBS threshold for early restoration. These findings support the use of material-specific adhesive strategies for vital pulp therapy and suggest that restoration timing should be considered together with cement type and adhesive strategy to optimize bonding outcomes. Further studies incorporating natural dentine substrates, advanced surface characterization techniques, and long-term aging protocols are warranted to confirm the clinical relevance of these findings.

## 4. Materials and Methods

### 4.1. Study Design

This in vitro factorial study examined three experimental factors: hydraulic calcium silicate-based cement type, adhesive strategy, and restoration interval. Shear bond strength (SBS) was defined as the primary outcome, while failure mode distribution was assessed as a secondary outcome. Two cements were investigated: MTA Angelus (Angelus, Londrina, PR, Brazil) and Well-Root PT (Vericom Co., Chuncheon, Republic of Korea). According to the manufacturers’ information, MTA Angelus is a powder–liquid material containing tricalcium silicate, tricalcium aluminate, calcium oxide, dicalcium silicate, and bismuth oxide. Well-Root PT is supplied as a premixed calcium aluminosilicate-based cement containing calcium aluminosilicate compound, zirconium oxide, calcium sulfate dihydrate, polyethylene glycol, and propylene glycol.

At each designated restoration interval (45 min, 24 h, or 7 days after cement placement), specimens were treated with one of three bonding protocols: Adper^TM^ Single Bond 2 (3M ESPE, St. Paul, MN, USA) using a two-step etch-and-rinse approach; Clearfil^TM^ SE Bond (Kuraray Noritake Dental Inc., Tokyo, Japan) using a two-step self-etch approach; or G-Premio Bond (GC Corp., Tokyo, Japan), a universal adhesive applied in self-etch mode.

Crossing the two cement types with the three adhesive strategies and three restoration intervals generated 18 experimental combinations in a 2 × 3 × 3 factorial design. The groups represented every combination of MTA Angelus or Well-Root PT with the two-step etch-and-rinse, two-step self-etch, or universal self-etch adhesive strategy at 45 min, 24 h, or 7 days. Fifteen specimens were assigned to each combination, yielding 270 specimens overall. Thus, each cement contributed 135 specimens, with 45 specimens allocated to each adhesive strategy before subdivision among the three restoration intervals. Group assignment followed a computer-generated randomization sequence established before specimen preparation.

**Figure 4:**
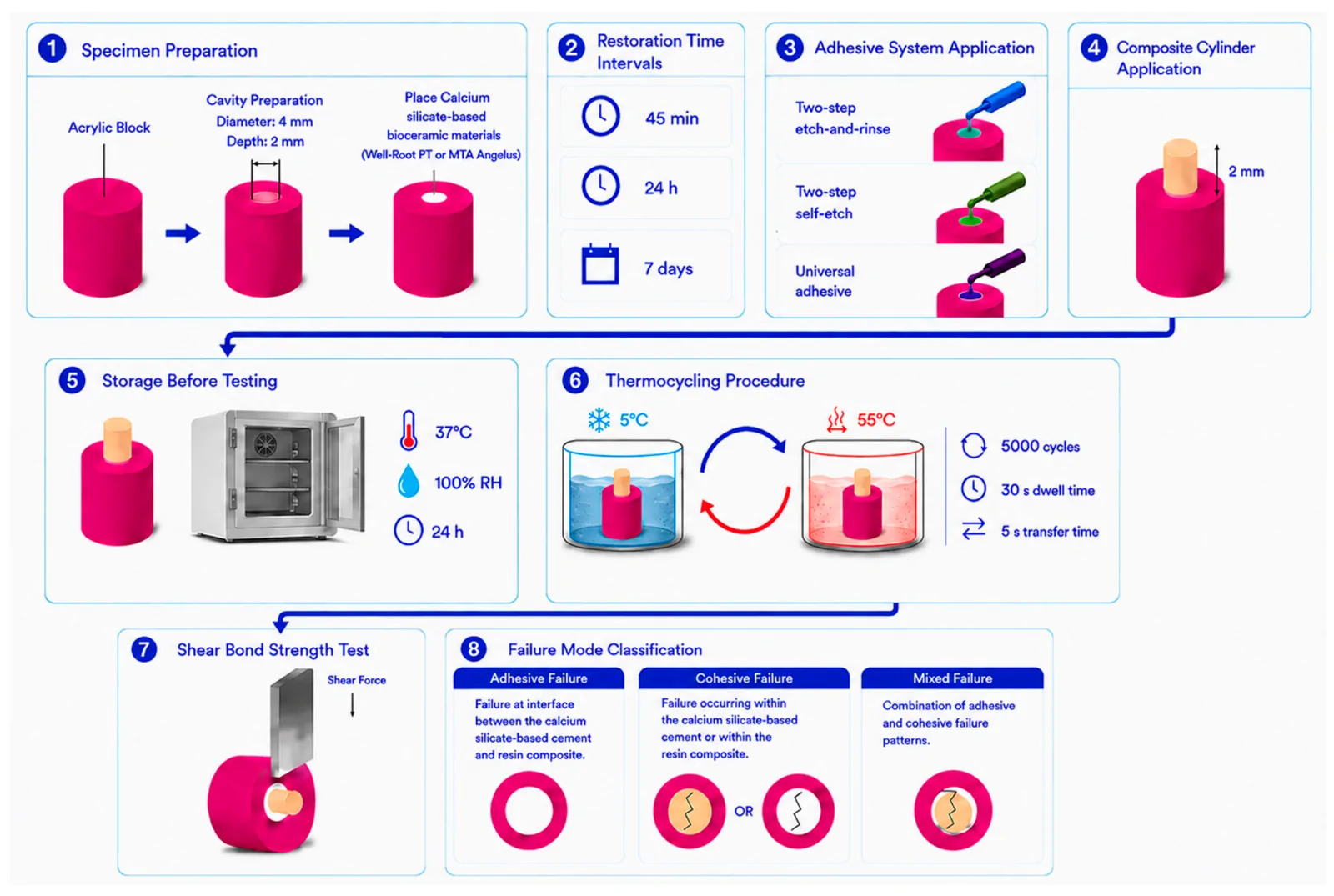
Schematic illustration of the experimental workflow. Acrylic blocks containing MTA Angelus or Well-Root PT were restored using three adhesive strategies at three restoration time intervals (45 min, 24 h, and 7 days). Resin composite cylinders were bonded to the cement surfaces, followed by shear bond strength testing and failure mode classification.

The complete experimental sequence, from specimen preparation and timed restoration to adhesive application, SBS testing, and failure mode assessment, is summarized in Figure 4.

### 4.2. Sample Size Calculation

Sample size requirements were estimated a priori using G*Power 3.1.9.7 (Heinrich Heine University, Düsseldorf, Germany). For the fixed-effects factorial ANOVA, the calculation assumed a medium effect size (f = 0.25), α = 0.05, 80% power, 18 experimental groups, and four numerator degrees of freedom for the highest-order interaction. These parameters indicated a minimum requirement of 197 specimens. To account for potential losses during preparation or testing, 15 specimens were included in each experimental combination, resulting in a final sample of 270.

### 4.3. Specimen Preparation

Cylindrical acrylic blocks were fabricated using self-curing acrylic resin. A standardized cylindrical cavity measuring 4 mm in diameter and 2 mm in depth was prepared at the center of each acrylic block. The dimensions of the cavities were verified using a digital caliper before material placement.MTA Angelus was prepared using the manufacturer-recommended powder-to-liquid ratio, whereas the premixed Well-Root PT was dispensed directly from its delivery system according to the manufacturer’s instructions. The respective cement was transferred into the prepared cavity with a hand instrument and adapted to the walls. A sterile flat plastic instrument was then used to level the material flush with the surrounding acrylic surface.During initial adaptation, a glass slab was placed gently over the filled cavity to obtain a standardized surface and reduce the likelihood of void formation, after which excess material was removed. Each specimen was covered with a moistened cotton pellet and maintained at 37 °C and 100% relative humidity until its designated restoration interval, providing standardized conditions for continued cement hydration.

### 4.4. Group Allocation and Restoration Time Intervals

Restoration was scheduled at 45 min, 24 h, or 7 days after placement of the calcium silicate-based cement. The earliest interval represented restoration following the setting period of the fast-setting materials, whereas the 24 h and 7-day conditions represented progressively later stages of cement hydration.At each time point, specimens were restored using one of the three adhesive strategies.

### 4.5. Adhesive Procedures

Before application of the adhesive systems, the moist cotton pellet was removed and the cement surface was examined visually. No polishing or mechanical surface treatment was performed. Any visible excess moisture was removed carefully with absorbent paper while avoiding complete surface desiccation.In the two-step etch-and-rinse group, 35–37% phosphoric acid (Scotchbond^TM^ Universal Etchant, 3M ESPE, St. Paul, MN, USA) was applied to the cement surface for 15 s. The surface was subsequently rinsed with distilled water and gently blot-dried before application and light activation of the adhesive in accordance with the manufacturer’s protocol.For the two-step self-etch protocol, the acidic primer was actively rubbed onto the cement surface as recommended by the manufacturer and then gently air-dried. A separate bonding resin was subsequently applied and light-cured.G-Premio Bond was applied solely in self-etch mode, with no preceding phosphoric acid treatment. The adhesive was distributed over the cement surface, air-thinned to facilitate solvent evaporation, and light-cured following the manufacturer-recommended protocol.Adhesive procedures were standardized by having a single operator perform them under uniform laboratory conditions.

### 4.6. Resin Composite Build-Up

A transparent Tygon tube (Saint-Gobain Performance Plastics, Akron, OH, USA), measuring 2 mm in internal diameter and 2 mm in height, served as a mold for the composite build-up. Following adhesive application, the tube was centered over the bonded area and aligned perpendicular to the cement surface.A nanofilled resin composite (Filtek^TM^ Z350 XT Universal Restorative, 3M ESPE, St. Paul, MN, USA) was placed into the tube with a composite instrument, taking care to minimize incorporation of air voids. Polymerization was performed for 20 s with an LED curing unit (Bluephase N, Ivoclar Vivadent, Schaan, Liechtenstein) delivering an irradiance of approximately 1200 mW/cm^2^. During light exposure, the curing tip was maintained perpendicular to the specimen and positioned close to the upper end of the tube. Irradiance was checked with a radiometer before each experimental session.After curing, the Tygon tube was slit and removed with a scalpel blade while avoiding lateral loading of the composite cylinder. Specimens showing visible defects, entrapped air, off-center composite build-up, or premature debonding were discarded and replaced.

### 4.7. Storage Before Testing

After composite build-up, all specimens were stored at 37 °C and 100% relative humidity for 24 h before SBS testing.

### 4.8. Thermocycling Procedure

Thermal aging comprised 5000 cycles between water baths maintained at 5 °C and 55 °C. Each cycle involved a 30 s dwell period at each temperature and a 5 s transfer between baths. The protocol was selected with reference to ISO TR 11405 recommendations for laboratory assessment of adhesive interfaces. SBS testing was performed after completion of the thermal cycling procedure.

### 4.9. Shear Bond Strength Test

For mechanical testing, each specimen was secured in a universal testing machine (Instron 3345, Instron Corp., Norwood, MA, USA). A stainless-steel knife-edge blade was aligned immediately adjacent to the cement–composite interface, and loading was applied at 1.0 mm/min until failure. The peak failure force (N) was converted to SBS (MPa) according to the following equation:SBS = F/A where F denotes the maximum failure load in Newtons and A the bonded area in mm^2^. For the 2 mm diameter composite cylinder, the bonded area was 3.14 mm^2^.

### 4.10. Failure Mode Analysis

Fractured interfaces were inspected at ×40 magnification with a stereomicroscope (Leica EZ4D, Leica Microsystems, Wetzlar, Germany). A single examiner blinded to group allocation categorized each specimen as adhesive when separation occurred at the cement–composite interface, cohesive when fracture occurred within either bonded material, or mixed when both fracture patterns were present.

### 4.11. Statistical Analysis

Statistical procedures were conducted in IBM SPSS Statistics 26.0 (IBM Corp., Armonk, NY, USA), with α set at 0.05. The distribution of SBS data was examined with the Shapiro–Wilk test, while equality of variances was assessed using Levene’s test. After confirming the assumptions for parametric testing, the main and interaction effects of cement type, adhesive strategy, and restoration interval were analyzed by three-way ANOVA. Tukey-adjusted pairwise comparisons were subsequently performed. Failure-mode frequencies were evaluated using Pearson’s chi-square or Fisher’s exact test, as appropriate.

## Figures and Tables

**Table 1:** Three-way ANOVA evaluating the effects of cement type, adhesive strategy, restoration timing, and their interactions on shear bond strength.

Source of Variation	df	F Value [-]	*p* Value [-]
Cement type	1	18.60	<0.001 *
Adhesive strategy	2	65.96	<0.001 *
Restoration time interval	2	35.51	<0.001 *
Cement × Adhesive	2	8.24	<0.001 *
Cement × Time	2	6.97	<0.001 *
Adhesive × Time	4	5.88	<0.001 *
Cement × Adhesive × Time	4	4.15	<0.001 *
Error	252	—	—

* Statistically significant at *p* < 0.05.

**Table 2:** Shear bond strength (SBS) values (MPa, mean ± SD) according to cement type, adhesive strategy, and restoration timing.

Adhesive Strategy	Time	MTA Angelus	Well-Root PT
Etch-and-rinse	45 min	5.80 ± 0.70 ^d^	7.36 ± 1.15 ^b^
Etch-and-rinse	24 h	9.66 ± 1.12 ^a^	9.14 ± 1.05 ^a^
Etch-and-rinse	7 days	8.76 ± 1.42 ^ab^	8.96 ± 1.15 ^a^
Two-step self-etch	45 min	5.15 ± 0.89 ^d^	6.79 ± 1.13 ^bc^
Two-step self-etch	24 h	7.55 ± 1.22 ^bc^	8.04 ± 1.37 ^ab^
Two-step self-etch	7 days	6.81 ± 1.05 ^c^	8.21 ± 1.67 ^ab^
Universal	45 min	3.88 ± 0.73 ^e^	5.47 ± 1.02 ^cd^
Universal	24 h	5.50 ± 1.13 ^d^	6.71 ± 1.39 ^bc^
Universal	7 days	5.27 ± 1.12 ^d^	6.57 ± 1.39 ^bc^

Values are expressed as mean ± SD in MPa. Superscript letters are based on Tukey-adjusted pairwise comparisons among the 18 experimental combinations. Different lowercase superscript letters indicate statistically significant differences among experimental groups. Means sharing at least one superscript letter are not significantly different, whereas means with no letters in common differ significantly (Tukey’s HSD, *p* < 0.05).

**Table 3:** Distribution of failure modes (adhesive/cohesive/mixed) according to adhesive strategy, restoration timing, and cement type.

Adhesive Strategy	Time	MTA Angelus	Well-Root PT
Etch-and-rinse	45 min	6/3/6	6/2/7
Etch-and-rinse	24 h	3/6/6	5/3/7
Etch-and-rinse	7 days	3/7/5	4/4/7
Two-step self-etch	45 min	8/2/5	7/2/6
Two-step self-etch	24 h	5/4/6	6/3/6
Two-step self-etch	7 days	5/5/5	5/3/7
Universal	45 min	11/1/3	10/1/4
Universal	24 h	8/2/5	8/2/5
Universal	7 days	8/3/4	7/2/6

Values are presented as adhesive/cohesive/mixed failure modes. Each group included 15 specimens.

## Data Availability

The data presented in this study are available from the corresponding author upon reasonable request.

## Abbreviations

CSC	Calcium silicate-based cement
MTA	Mineral trioxide aggregate
VPT	Vital pulp therapy
SBS	Shear bond strength
SD	Standard deviation
ANOVA	Analysis of variance
LED	Light-emitting diode
SEM	Scanning electron microscopy
EDX	Energy-dispersive X-ray spectroscopy
RH	Relative humidity
